# Prevalence and Comorbidities of Tourette Syndrome in Children and Adolescents: Insights from the National Survey of Children’s Health (NSCH)

**DOI:** 10.1192/j.eurpsy.2025.450

**Published:** 2025-08-26

**Authors:** M. Saeidi, O. Alzein, K. Jafari, M. Salehi, S. Jaka, S. Gunturu

**Affiliations:** 1 Department of Psychiatry, Bronx Care Health System, New York; 2 Department of Psychiatry, University of Minnesota School of Medicine, Minneapolis; 3 Department of Psychiatry and behavioral Sciences, Nassau University of Medical Center; 4 Department of Psychiatry, Icahn School of Medicine at Mount Sinai, New York, United States

## Abstract

**Introduction:**

Tourette Syndrome (TS) is a neurodevelopmental disorder involving multiple motor and vocal tics, typically beginning in childhood with symptoms peaking around age 10-12. Though the exact cause remains unknown, genetic and environmental factors are believed to contribute to its development. Children with TS often face significant social, emotional, and academic challenges, further complicated by comorbidities which can lower their quality of life and place stress on family dynamics.Using data from the 2021 National Survey of Children’s Health(NSCH), this study provides updated insights into the prevalence and comorbidities of TS.

**Objectives:**

Assess TS prevalence in children aged 3-17 using 2021 data

Identify common TS comorbidities and evaluate TS’s demographic distribution

**Methods:**

This cross-sectional study utilized 2021 NSCH data collected via parent-proxy responses in English and Spanish through mail and web-based surveys in the United States. Statistical analyses, including t-tests, Chi-Square tests, and multivariate regression, were performed to explore associations between TS, socio-demographic factors, and comorbidities. Results were reported using adjusted odds ratios and confidence intervals, with analyses conducted in Stata 18.0.

**Results:**

This study included 79,236 participants, of which 208 (0.26%) had TS.The TS group had a mean age of 12.7 years, with most cases found in adolescents aged 11-17.Males made up 74% of TS cases. While TS was slightly more common in White participants (72%), no significant racial differences were found. Higher-income households had lower odds of TS.Comorbidities were more common in individuals with TS:

Neurodevelopmental and Behavioral Disorders: ADHD (49% in TS vs. 10.2% in non-TS), Autism (21% vs. 3.2%), Learning disabilities (28% vs. 7.5%), Developmental delay (23% vs. 6%), Behavioral problems (36%)

Psychiatric Disorders: Anxiety (60% in TS vs. 11.3%), Depression (25% vs. 5%), Seizures (6%), Headaches (14.4%).

Immune Conditions: Asthma (15.5% in TS vs. 8%), Allergies (37%).

Physical Health: Hearing problems (3.8%), Vision problems (4.3%). *Fig. 1*

Children with TS born prematurely or with low birth weight had higher rates of comorbidities. Asthma was more common in the TS with a history of prematurity (37.1%) and TS with a history of low birth weight(30.7%) compared to the overall TS population (15.5%). Autism was also more prevalent in these groups, suggesting increased risks of comorbidities due to premature birth and low birth weight. *Fig.2*

**Image 1:**

**Figure:**
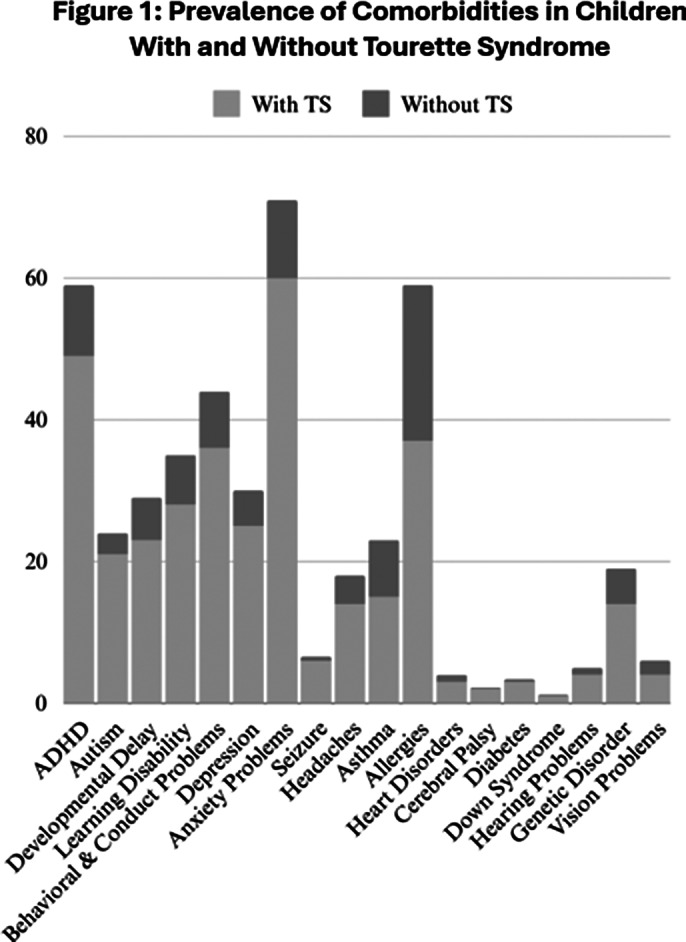

**Image 2:**

**Figure:**
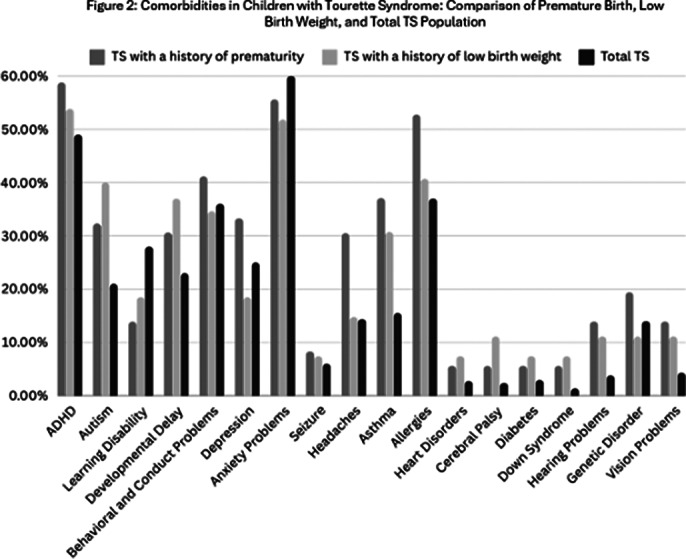

**Conclusions:**

The study highlights significant socio-demographic disparities and the increased burden of comorbid conditions in children with TS. These findings emphasize the need for early diagnosis and comprehensive management strategies to address the complex challenges of TS, particularly the high prevalence of neurodevelopmental, psychiatric, and physical health comorbidities.

**Disclosure of Interest:**

None Declared

